# MXene-Carbon Nanotube Composites: Properties and Applications

**DOI:** 10.3390/nano13020345

**Published:** 2023-01-14

**Authors:** Fatemeh Mohajer, Ghodsi Mohammadi Ziarani, Alireza Badiei, Siavash Iravani, Rajender S. Varma

**Affiliations:** 1 Department of Organic Chemistry, Faculty of Chemistry, Alzahra University, Tehran 19938-93973, Iran; 2 School of Chemistry, College of Science, University of Tehran, Tehran 14179-35840, Iran; 3 Faculty of Pharmacy and Pharmaceutical Sciences, Isfahan University of Medical Sciences, Isfahan 81746-73461, Iran; 4 Institute for Nanomaterials, Advanced Technologies and Innovation (CxI), Technical University of Liberec (TUL), 1402/2, 461 17 Liberec, Czech Republic

**Keywords:** MXenes, carbon nanotubes, electromagnetic interference shielding, sensing, catalysis

## Abstract

Today, MXenes and their composites have shown attractive capabilities in numerous fields of electronics, co-catalysis/photocatalysis, sensing/imaging, batteries/supercapacitors, electromagnetic interference (EMI) shielding, tissue engineering/regenerative medicine, drug delivery, cancer theranostics, and soft robotics. In this aspect, MXene-carbon nanotube (CNT) composites have been widely constructed with improved environmental stability, excellent electrical conductivity, and robust mechanical properties, providing great opportunities for designing modern and intelligent systems with diagnostic/therapeutic, electronic, and environmental applications. MXenes with unique architectures, large specific surface areas, ease of functionalization, and high electrical conductivity have been employed for hybridization with CNTs with superb heat conductivity, electrical conductivity, and fascinating mechanical features. However, most of the studies have centered around their electronic, EMI shielding, catalytic, and sensing applications; thus, the need for research on biomedical and diagnostic/therapeutic applications of these materials ought to be given more attention. The photothermal conversion efficiency, selectivity/sensitivity, environmental stability/recyclability, biocompatibility/toxicity, long-term biosafety, stimuli-responsiveness features, and clinical translation studies are among the most crucial research aspects that still need to be comprehensively investigated. Although limited explorations have focused on MXene-CNT composites, future studies should be planned on the optimization of reaction/synthesis conditions, surface functionalization, and toxicological evaluations. Herein, most recent advancements pertaining to the applications of MXene-CNT composites in sensing, catalysis, supercapacitors/batteries, EMI shielding, water treatment/pollutants removal are highlighted, focusing on current trends, challenges, and future outlooks.

## 1. Introduction

**Figure 1:**
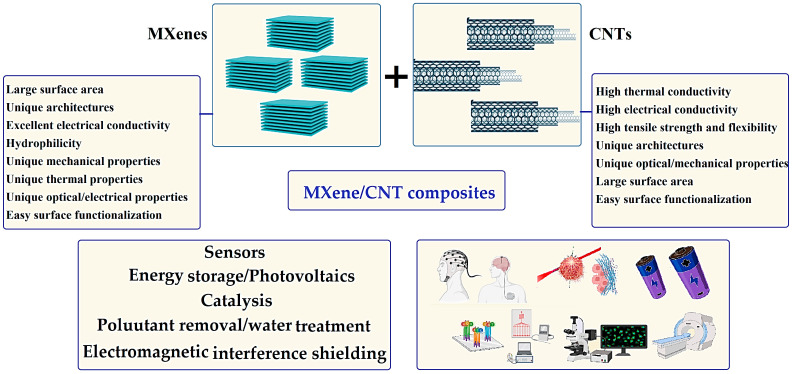
MXene-CNT composites with alluring potentials.

MXenes as new celebrated materials with unique electronic, optical, thermal, mechanical, magnetic, and electrochemical characteristics have garnered considerable research interest, opening great opportunities for (photo)catalysis, lithium (Li)-sulfur (S), and sodium-ion batteries, electromagnetic interference (EMI) shielding, and supercapacitors, as well as tissue engineering, drug delivery, cancer (nano)theranostics, and regenerative medicine [1,2,3,4,5,6,7]; these materials have shown excellent optoelectronic properties as well as high metallic conductivity and optical transmittance, which make them promising candidates for photovoltaic systems [8,9]. MXenes with general formula of M_n+1_X_n_T_x_ (*n* = 1–3) have been synthesized using a wide variety of techniques, including etching processes [10], electrochemical synthesis [11], electrochemical fabrication [12], the urea glass route [13], microwave-assisted synthesis [14], chemical vapor deposition [15], hydrothermal production [16], ultrasonic-assisted synthesis [17], atomic layer deposition [18], among others. On the other hand, CNTs with suitable interfacial area, multifunctionality, and needle-like shapes have been fabricated using carbon arc discharge, spray pyrolysis, chemical vapor deposition, flame synthesis, laser-ablation, etc. [19,20]. The unique properties of CNTs such as good heat conduction, tensile strength, flexibility, hollow monolithic structures, suitable penetrability, and optico-electrical features make them promising candidates in environmental sciences and bio- and nanomedicine domains [21,22]. However, there are still important challenges regarding the flexibility, functionality, and stability of MXenes; thus, improvements are still required, particularly by applying hybridization techniques, suitable functionalization/modification, and optimization of synthesis/reaction conditions [23,24,25,26,27,28]. In this context, different types of MXene-based composites have been fabricated, such as MXene-polymer and MXene-oxide composites [29,30,31,32]. One of the recently introduced hybridized composites is the MXene-carbon nanotube (CNT) structure [33], with excellent electrochemical performances and unique mechanical properties (Figure 1). These hybrid composites pave the way toward the synthesis of composites with different sensing, catalysis, water treatment, pollutant removal, EMI shielding, and supercapacitor/battery applications [1,34,35,36,37,38].

The hybridization of MXenes with CNTs can improve their mechanical properties and alkaline resistance [39]. For instance, three-dimensional (3D) nano-fillers of MXene (Ti_3_C_2_T_x_)/CNTs were constructed with high stability and enhanced interfacial adhesion between adjacent fiber yarns along with the increased tensile and flexural strength, showing excellent surface roughness and flexural strength even after 120 days of immersion in alkali solutions [39]. Moreover, interfacial adhesion could be enhanced in MXene-based membranes via the hybridization with CNTs, wherein the robust π–π interaction and van der Waals forces of CNTs impel the close-fitting of MXene nanosheets to improve anti-swelling features and interfacial bonding forces [40]. It has been revealed that CNTs with the effects of confined-space mass transfer and excellent mechanical strength could improve the performance of MXene-based membranes (~5 times) after hybridization, showing great potential in resource recovery and energy-saving [40]. In addition, MXene-CNT (nano)composites exhibit enhanced electrochemical features and can be applied directly in designing electrodes in supercapacitors [41]. Li et al. [41] reported the preparation of MXene-CNT nanocomposite paper, which could be employed in Li ion batteries with improved cycling stability and high capacitance [41]. Herein, the most recent advancements regarding the properties and applications of MXene-CNT composites are cogitated, focusing on recent trends, crucial challenges, and future directions. The versatile applications of MXene-CNT hybrid composites in the field of catalysis/electrocatalysis, energy storage, sensors, EMI shielding, and pollutant removal/water treatment are discussed. Hopefully, this review will encourage researchers for further comprehensive studies on such composites with fascinating properties and multifunctionality. Other dimensions of the applications of such materials, especially in biomedicine, energy storage, soft robotics, electronic skins, and smart/wearable sensors still require multidisciplinary research for industrialization/commercialization.

## 2. MXene-CNT Composites

A variety of MXene-CNT composites has been designed with versatile applications in catalysis, electrocatalysis, EMI shielding, energy storage, water treatment, sensors, etc. (Table 1) [29,30,31,32,42]. These composites have been synthesized using mechanical mixing, self-assembly, co-dispersion, electrophoretic deposition, in-situ growth of CNTs on MXenes by chemical vapor deposition, thermal treatment, and microwave-assisted and hydrothermal processes. Among several MXene-CNT architectures introduced, aerogels and foams with 3D structures can be considered as attractive candidates with unique mechanical properties and excellent permeability for gas/liquid, offering great opportunities for various applications [33,43]. However, future studies ought to focus on systematic analysis of thermal, mechanical, porosity, and electrical/electronic features of MXene-CNT composites. It appears that the synergistic effects of MXenes and CNTs in hybrid structures can reduce/prevent the severe stacking issues of two-dimensional (2D) structures, thus improving the properties for different applications [33,44].

### 2.1. Sensing

MXenes have shown fascinating electronic, optical, mechanical, and thermal features, which make them suitable candidates for hybridization with other materials to provide suitable (nano)composites with versatile diagnostic applications [3,29,76,77,78,79]. Because of their excellent potential in electrochemical sensing, nanohybrids of MXene (Ti_3_C_2_T_x_) nanoribbons/CNTs were constructed for the modification of glassy carbon electrode as sensing platforms, offering a new strategy without electrodeposition for specific detecting Hg^2+^ [80]. MXenes have displayed excellent spontaneous adsorption and reduction potential towards Hg^2+^, causing the self-reduction of Hg^2+^ to be pre-concentrated on the electrode surfaces. Notably, the electronic features of CNTs could help to provide an electrodeposition-free and sensitive sensor with robust sensing activities for analyzing Hg^2+^ (the LOD was ~5.2 nM, and the linearity range was ~0.01–7.0 μM) [80].

**Figure 2:**
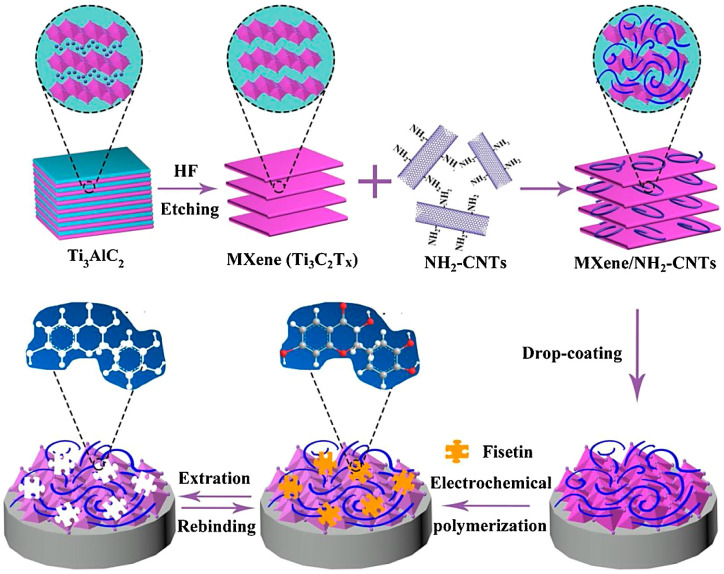
The preparative process of molecularly imprinted polymer (MIP)/MXene/amino (NH_2_)-CNTs/glass carbon electrode (GCE) for specific sensing of fisetin, along with the related adsorption mechanisms. Adapted from Ref [81] with permission. Copyright 2020 Elsevier.

An electrochemical sensor based on hierarchical porous MXene/amino CNT composites and the benefits of the molecularly imprinted method were synthesized with suitable stability for the specific detection of fisetin (the LOD was ~1.0 nmol L^−1^, and the linearity range was ~0.003–20.0 μmol L^−1^) (Figure 2). The extraction time, pH of the supporting electrolyte, incubation time, the ratio of functional monomer to template molecule, and the polymerization cycles are important factors to increase the sensitivity/selectivity of this type of sensor [81]. Similarly, an electrochemical biosensor was designed from MXene (Ti_3_C_2_T_X_) and multi-walled CNTs to provide composite encompassing molecularly imprinted polymers for the sensitive detection of amyloid-β protein. The sensor with advantages of stability, repeatability, reproducibility, and reusability could successfully detect this protein with a linear range of 1.0–100.0 fg mL^−1^ and an LOD of 0.3 fg mL^−1^ [82]. Ni et al. [83] designed a dopamine electrochemical sensor with high sensitivity from MXene (Ti_3_C_2_), graphitized multi-walled CNTs, and ZnO nanospheres. Accordingly, the sensor displayed significant sensitivity (16 A/M) with an LOD of ~3.2 nM and a wide linear range (0.01–30 μM) under the optimal experimental conditions. The sensor exhibited improved stability and anti-interference potential as well as suitable accuracy in human serum samples, opening new opportunities for designing smart electrochemical sensors. Despite the importance of synthesis conditions and functionalization processes, the optimization of reaction and experimental conditions is also vital to obtain high yields and improved results [83]. Xia et al. [84] reported the design of molecularly imprinted polymer film through the electropolymerization of 1 H-pyrrole-3-carboxylicacid in the presence of diethylstilbestrol on CNTs/Cu_2_O nanoparticles/MXene (Ti_3_C_2_T_x_)-modified electrodes. The application of MXenes with accordion-like structures provided suitable electrical conductivity and enabled the immobilization of Cu_2_O nanoparticles. On the other hand, CNTs were deployed for improving the sensitivity of these electrochemical sensors with a wide linear response range (0.01–70 μM) and an LOD of 6 nM, providing suitable sensors with good stability for the reliable detection of diethylstilbestrol (a nonsteroidal estrogen medication) [84].

Designing intelligent sensors for skin and healthcare monitoring with enough flexibility is one the important fields of science in wearable electronics, helping to evaluate the physical signals from the human body. In this respect, challenges regarding their sensitivity, stability, and working range still persist [85]. For instance, to design sensors for wearable healthcare monitoring, CNT/Ti_3_C_2_T_x_/polydimethylsiloxane composites were constructed [86]. The sensor exhibited reliable responses at various frequencies and long-term cycling durability (>1000 cycles), as well as the benefits of excellent anti-interference to temperature alteration and water washing. This sensor with suitable applicability for monitoring human joint motions could be also deployed for real-time monitoring of the electrocardiogram (ECG) signals and joint movements [86]. In another study, a foam-shaped strain sensor was designed using MXene (Ti_3_C_2_T_x_), multi-walled CNTs, and thermoplastic polyurethane [85]. Accordingly, this sensor displayed a wide working strain range of ~100% with high sensitivity, showing excellent gas permeability with an appropriate elastic modulus close to that of skin, making it suitable to be applied as a wearable sensor. Subtle and large human movements could be detected, along with gesture recognition, offering a flexible and wearable sensor [85]. Cai et al. [87] introduced a flexible strain sensor constructed from MXene (Ti_3_C_2_T_x_) and CNTs with excellent electric features consisting of 2D MXene nano-stacks and conductive/stretchable one-dimensional (1D) CNT crossing; the sensor exhibited significant stretchability (up to 130%) and sensitivity along with a tunable sensing range (30–130% strain), showing great reliability and stability (more than 5000 cycles). Such sensors can be employed for real-time monitoring and in situ analyzing of physiological signals with health and sporting purposes [87].

**Figure 3:**
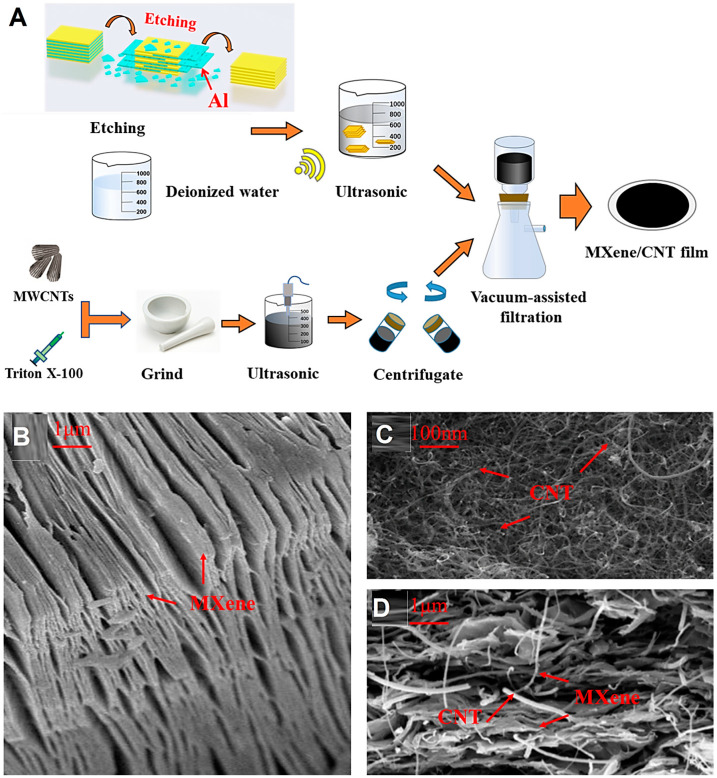
(**A**) The preparative process of sandwich-like MXene/CNT films. (**B**) Scanning electron microscopy (SEM) images (cross-section) of pure MXene (Ti_3_C_2_T_x_). (**C**) SEM of CNTs. (**D**) SEM of MXene/CNT film (cross-section). Adapted from Ref [88] with permission. Copyright 2021 Elsevier.

With developments in wearable electronics, designing sensors with multifunctionality and high performance to monitor abilities is one the important challenges at the industrial scale. In one study, hybrid composites of multi-walled CNTs and MXenes were designed for lifetime health monitoring applications via a simple mixing and spray vacuum filtration technique (Figure 3) [88]. The composite films exhibited good stability to respond to changes in the resin state; after 1000 cycles of fatigue evaluations, the hybrid film sensors maintained a good response and consistency in response to the external force for a long time. These film sensors with a long service time, significant response sensitivity, excellent synchronization to stretching, and solid real-time function could successfully monitor gel spots with various positions and thicknesses in real time. However, more explorations are still required using modern and cutting-edge technologies to adjust the bonding between MXene/CNT film sensors and composite laminates. Notably, the stretchability and flexibility of these film sensors can still be improved by optimization processes [88]. In addition, a wearable 3D porous polyurethane sponge sensor was designed deploying MXene/CNT composites constructed through a simple ultrasonic dip-coating technique [89]. The application of these composites could improve the measurement capability of the sensor from a wide-compressive strain range (−80%) to a wide-stretching strain range (60%), providing a sensor with great electrical response and stability (>5000 cycles) to detect vocal vibrations, human movements, subtle expressions, etc. [89].

**Figure 4:**
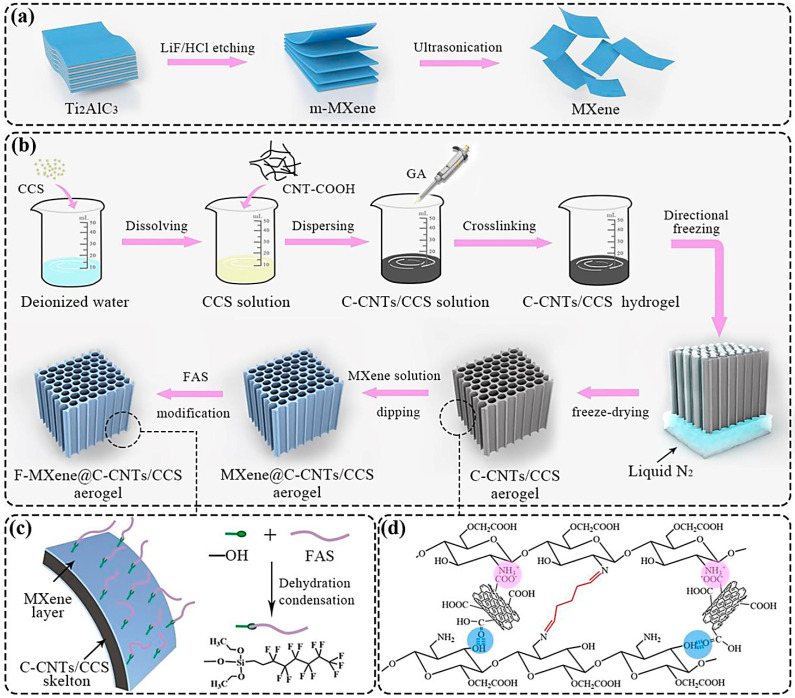
(**a**,**b**) The preparative process of MXene (Ti_3_C_2_T_x_) nanosheets and an MXene-coated carboxylated (C)-CNTs/carboxymethyl chitosan (CCS) composite aerogel. (**c**) The chemical modification of MXene layers by 1H,1H,2H,2H-perfluorooctyltriethoxysilane (FAS). (**d**) The generation of imine bonds between the amino groups of CCS and the aldehyde groups of glutaraldehyde (GA) as cross-linkers with related interactions between carboxylated CNTs and CCS. Adapted from Ref [90] with permission. Copyright 2021 Elsevier.

MXene-coated carboxylated CNTs/carboxymethyl chitosan aerogels were designed with superb electrical stability and repeatability for sensing piezo-resistive pressure (Figure 4) [90]. The designed sensor exhibited a fast response time (62 ms) and a wide detection range (up to 80 kPa) owing to the synergism of the dual-conductive MXene-CNT network. It could be employed for the sensitive monitoring of human motions such as joint movements, walking, finger tapping, running, and pronunciation detection [90]. Chen et al. [91] reported the design of conductive films using dendritic–lamellar MXene/carbon nanotube/polyvinylpyrrolidone electrodes, providing flexible composites for wearable tactile sensors and artificial skin. As a result, the LOD was ~0.69 Pa with a response time of ~48 ms after the evaluation of the sensor for pulse measurement and voice recognition [91].

### 2.2. Wastewater Treatment/Remediation and Pollutants Removal

**Figure 5:**
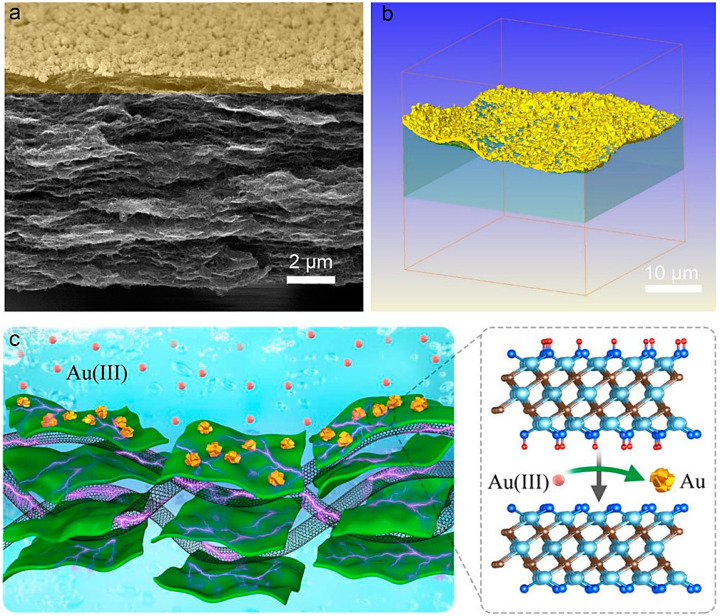
(**a**) SEM image of MXene/CNT-Au membrane (cross-sectional image). (**b**) Three-dimensional (3D) X-ray tomography (XRT) image of the membrane. (**c**) The proposed redox reaction mechanism of Au (III) rejection by the MXene-CNT hybrid membrane. Adapted from Ref [93] with permission. Copyright 2020 American Chemical Society.

MXenes (Ti_3_C_2_T_x_) intercalated with CNTs were designed to provide thin-film nanocomposite membranes with a superb degree of cross-linking and roughness [92]. Compared to the composite membranes without interlayered structures, the prepared membranes exhibited a water flux four times higher and a lower specific salt flux. These membranes could be efficiently deployed for wastewater treatment with improved rejection of ammonia nitrogen [92]. An MXene (Ti_3_C_2_T_x_)-CNT hybrid membrane was designed with robust potential for capturing precious metal ions from solutions (Figure 5) [93]. As a result, gold (Au) ions (~99.8%) could be obtained from a solution with an extremely low concentration of 20 ppm. The considerable redox activity of C–Ti–OH could be the reason for this excellent precious metal trapping potential, paving a suitable way for recovery/isolation of precious metal ions from wastewaters [93].

MXene/CNT/cotton fabric composites with water transport features, strong optical absorption, and light-to-thermal conversion have been prepared through a layer-by-layer assembly to be applied as solar steam formation for textile wastewater purification [94]. Under sun illumination, the evaporation rate reached 1.35 kg m^−2^ for water and >1.16 kg m^−2^h^−1^ for textile wastewater, providing promising candidates for wastewater purification by solar-evaporation [94]. A variety of lamellar membranes has been designed using 2D nanomaterials with excellent potential for molecular separation with high efficiency [95]. In one study, high-performance hetero-structured membranes with fusiform transport channels were constructed using MXene layers and CNTs, showing significant water permeation (1270 L m^−2^ h^−1^ bar^−1^) [95]. Sun et al. [96] designed multidimensional MXene-CNT ultrathin membranes with high efficiency and stability owing to the van der Waals interactions (hydrogen bond) and repulsion forces between MXene and CNTs, showing improved permeability with a distinct suppressed swelling feature through a thermal cross-linking route. These high-performance membranes can be considered as promising candidates for water purification [96].

Thirumal et al. [97] reported the synthesis of MXene-CNTs by applying a catalytic chemical vapor deposition technique. These composites with significant photocatalytic performance were deployed for the efficient elimination of rhodamine B dye pollutant. Compared to the pure MXenes for photocatalytic dye degradation (the efficiency was 60%), MXene-CNT hybrids exhibited improved efficiency of ~75%. It appears that the introduced chemical vapor deposition technique can be applied for designing novel MXene-CNT hybrid structures with a high yield of production after the optimization process, providing suitable composites with good stability (recyclability) for the removal of hazardous pollutants [97]. For the removal of pharmaceutical wastes, MXene-CNT composites were fabricated through the pyrolysis of homologous metal-organic framework precursors, showing catalytic performance for the degradation of antibiotic tetracycline hydrochloride. Notably, these composites exhibited a high specific surface area with improved adsorption capacity (~35.9%) and degradation rate (0.26 min^−1^) [98].

### 2.3. EMI Shielding Performance

**Figure 6:**
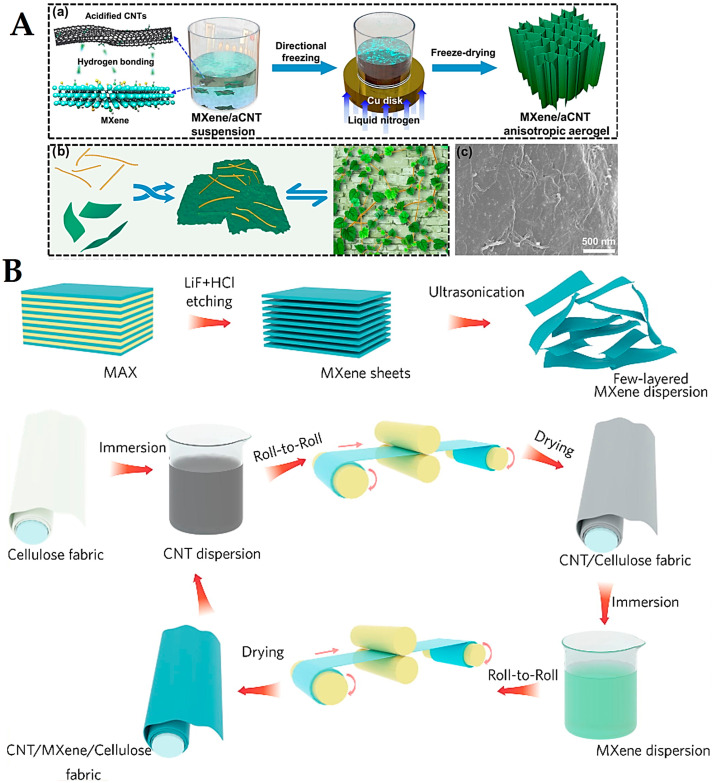
(**A**-**a**) The preparative process of MXene/CNT aerogels. (**b**) Bionic assembly between MXene nanosheets and CNTs. (**c**) SEM image of MXene/acidified CNT anisotropic aerogels with biomimetic microstructures. Adapted from Ref [99] with permission. Copyright 2021 American Chemical Society. (**B**) Roll-to-roll layer-by-layer assembly technique for fabricating bark-shaped CNT/MXene textiles with good EMI shielding performance. LiF: lithium fluoride. Adapted from Ref [101] with permission. Copyright 2021 Elsevier.

MXenes have shown attractive hydrophilicity and electrically conductive transition, making them suitable for electrically conductive and EMI shielding purposes [99]. However, it is difficult to construct compressible three-dimensional (3D) architectures with significant conductivity from MXenes due to the weak interaction among MXene nanosheets. In one study, MXene (Ti_3_C_2_T_x_)/acidified CNT anisotropic aerogels with high stability were designed, inspired by the plant (*Parthenocissus tricuspidata*), showing super-elasticity and considerable thermal insulation (Figure 6A) [99]. The robust conductivity (447.2 S m^−1^) and ultralow density (9.1 mg cm^−3^) demonstrated a robust EMI shielding efficiency of ~51 dB at an ultralow filler content of 0.3 vol %; by enhancing the density of composites to 18.2 mg cm^−3^, the EMI shielding effectiveness could reach 90 dB [99]. Weng et al. [100] constructed MXene (Ti_3_C_2_T_x_)-CNT composite films with high stability, flexibility, and conductivity using a spin spray layer-by-layer technique for EMI shielding purposes. These films exhibited significant conductivity (up to 130 S cm^−1^) with high specific shielding effectiveness (up to 58 187 dB cm^2^ g^−1^), paving a way for next-generation EMI shielding with improved absorption and electrical conductivity [100]. In addition, MXene (Ti_3_C_2_T_x_)-CNT hybrid composites were combined with a waterborne polyurethane matrix for EMI shielding and sheet heater purposes [35]. The composite films exhibited significant electrical conductivity with suitable mechanical flexibility, providing EMI shielding activity (~20–70 dB), along with heat dissipation and excellent Joule heating functions. Such composites with superb EMI shielding and thermal control capabilities can be considered as promising materials in designing wearable electronic devices [35]. In addition, bark-shaped CNT/MXene (Ti_3_C_2_T_x_) composite films were constructed using a roll-to-roll layer-by-layer assembly tactic (Figure 6B) [101]. After that, these films were decorated on the surfaces of fibers to obtain composites with suitable air permeability, electrical conductivity, and flexibility. These composites with improved electro-thermal activity and multi-interface scattering influences exhibited EMI shielding performance, along with significant sensitivity as the flexible piezoresistive sensors to monitor the human movements/motions, paving the way for designing EMI shielding materials and smart wearable electronics [101]. Moreover, the functionalization could increase the shielding by 15.4%, wherein MXene-CNT composites were designed for EMI shielding purposes; the composites could block 99.99% of the electromagnetic radiation. They had improved thermal stability along with a maximum electric conductivity of ~12.5 Scm^−1^ [102].

In an impressive study, 3D porous hybrid aerogels were constructed from MXene (Ti_3_C_2_T_x_) and CNTs by applying a freezing technique, showing suitable applicability for lightweight EMI shielding [103]. As was indicated, the synergistic effects from the lamellar and porous structures of the aerogels could extensively contribute to their fascinating electrical conductivity (9.43 S cm^−1^) along with the excellent value of EMI effectiveness (~103.9 dB at 3 mm thickness) at the X-band frequency. The incorporation of CNTs in these hybrid aerogels could significantly enhance the mechanical robustness and improve the compressional modulus compared to the pristine MXene aerogels [103]. For both EMI shielding and solar-thermal conversion applications, polymer-based nacre-like conductive films were constructed from hybrid MXene-CNT structures, wherein single-walled CNTs with unique electrical conductivity along with the fire/heat resistance and MXenes with lamellar and highly oriented structures were deployed to provide a significant electrical conductivity of ~1851.9 S cm^−1^ [104]. These flexible composite films with long-term environmental stability and structural integrity provided superb EMI shielding effectiveness (~78.9 dB) and considerable specific shielding effectiveness (~15,263.1 dB cm^2^ g^−1^); they could be applied for solar-thermal energy conversion with high performance [104].

### 2.4. Catalysis

**Figure 7:**
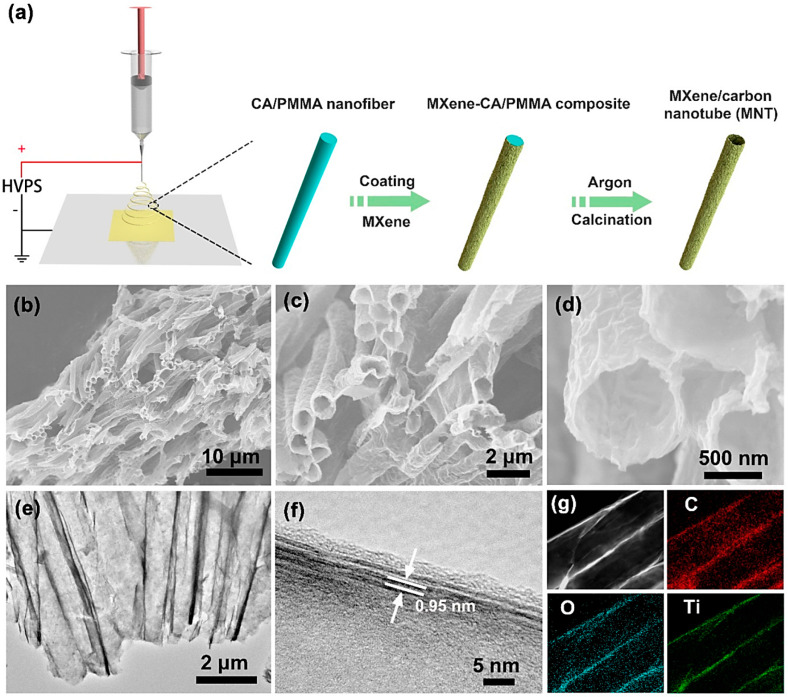
(**a**) The preparative process of MXene/CNT composites, with (**b**–**d**) SEM images and (**e,f**) high resolution transmission electron microscopy (HR-TEM) images. (**g**) The elemental mapping demonstrating the carbon (C), oxygen (O), and titanium (Ti) distributions of the composite. Adapted from Ref [110] with permission. Copyright 2021 American Chemical Society.

MXenes have been employed to develop a variety of (nano)catalysts with environmental and biomedical applications [6,36,105,106,107,108]. For instance, MXene@Pt/single-walled CNTs were constructed as nanocatalyst hydrogen evolution reactions. As a result, these catalysts exhibited ultrahigh stability with a high-volume current density of up to 230 mA cm^−3^ at −50 mV versus a reversible hydrogen electrode and a low over-potential of −62 mV versus a reversible hydrogen electrode at the current density of −10 mA cm^−2^ [109]. On the other hand, to obtain a continuous power supply and to generate appropriate chemicals (e.g., carbonates), the electrochemical carbon dioxide conversion at ambient temperature can be considered as a promising strategy [110]. With this purpose, parallel-aligned tubular MXene (Ti_3_C_2_T_x_)/CNT composites were designed via a self-sacrificial templating technique with significant catalytic activity and high stability, promoting the adsorption of CO_2_ and accelerating the decomposition of lithium carbonate (Figure 7) [110]. In addition, MXene (Ti_3_C_2_T_x_)-CNT composites were designed with remarkable electrical conductivity and corrosion resistance as supporting materials for Pt [36]. Compared to the commercial Pt on carbon catalysts, the introduced catalyst could enhance the durability and improve the oxygen reduction reaction performance. The composite could be applied as a cathode catalyst for a single cell and stack, and the maximum power density of the stack reached 138 W [36].

Cobalt (Co)-tipped CNT/MXene (Ti_3_C_2_) composites were constructed using a metal-organic-framework-engaged tactic for oxygen reduction reaction applications [111]. These composites exhibited suitable oxygen reduction reaction due to the abundant Co–N/C active sites and rationally significant graphitization of carbon, along with high surface areas. In addition, superb stability could be obtained compared to the commercial platinum (Pt)-based electrocatalysts, showing excellent potential for renewable conversion and storage applications [111]. In another study, MXenes (Ti_3_C_2_T_x_) were hybridized with Co/N-CNTs to provide bifunctional electrocatalysts for oxygen reduction and oxygen evolution reactions. The robust interfacial coupling and electron transfer could be detected, facilitating the electrocatalytic performances of these composites towards these reactions in alkaline solution [112]. Yang et al. [113] introduced multiwall CNT-based composites loaded with MoS_2_ and MXene (Ti_3_C_2_T_x_) quantum dots with suitable electrocatalytic activities for oxygen reduction and methanol oxidation reactions in alkaline solution. The catalysts demonstrated superb oxygen reduction reaction performance along with electro-oxidation activity for methanol in alkaline solution, providing a maximum methanol oxidation current density at 2.2 V of 160 A g^−1^ [113]. Moreover, NiCoFe-layered double hydroxide/MXene (Ti_3_C_2_)/N-doped CNT structures were designed with optimum nitrogen content, strong electronic interactions, high surface area, plentiful active sites, and improved electrical conductivity, showing significant electrocatalytic performance towards oxygen evolution and oxygen reduction reactions. These composites can be considered as high-performance bifunctional catalysts for oxygen electrocatalytic reactions in metal–air batteries [114].

### 2.5. Supercapacitors and Batteries

**Figure 8:**
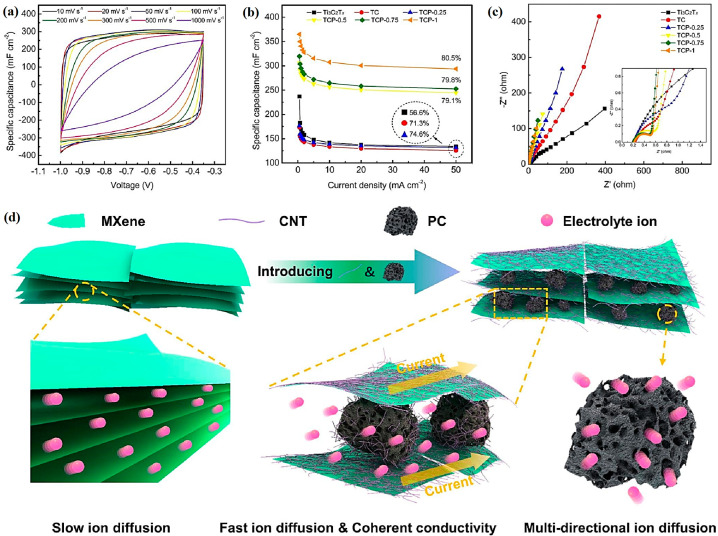
(**a**) Cyclic voltammetry profiles of MXene (Ti_3_C_2_T_x_)/CNT/porous carbon (TCP) films at various scan rates. (**b**) The rate potentials based on galvanostatic charge–discharge profiles. (**c**) Nyquist plots. (**d**) The ion transport in MXenes and TCP films. PC: porous carbon. Adapted from Ref [116] with permission. Copyright 2021 Elsevier.

MXenes with robust electrical conductivity have attracted special attention as electrode materials in designing supercapacitors [115]. In one study, MXene (Ti_3_C_2_T_x_)/CNT composite films were designed with outstanding functions as supercapacitor electrodes, showing significant capacitance of 300 F g^−1^ at 1 A g^−1^ with a superior rate of performance of 199 F g^−1^, even at 500 A g^−1^, along with high stability (~92% capacitance retention after 10,000 cycles at 20 A g^−1^). These MXene/CNT composites can be employed for designing flexible, portable, and highly integrated supercapacitors [115]. In addition, CNTs were employed for constructing highly conductive net structures, tightly anchoring porous carbon on MXene flakes, and causing fast electron delivery by enhancing the contact area between MXene and porous carbon (Figure 8) [116]. Accordingly, MXene (Ti_3_C_2_T_x_)/CNT/porous carbon films with suitable flexibility were designed to provide high areal specific capacitance of 364.8 mF cm^−2^ at 0.5 mA cm^−2^ (>80% even at a high current density of 50 mA cm^−2^). In addition, the designed supercapacitor exhibited a large areal energy density of 10.5 μ Wh cm^−2^ at 29.8 μ W cm^−2^, showing excellent potential of MXene-CNT hybrid composites for designing supercapacitors with significant rate capabilities and large charge storage capacities [116]. Cai et al. [117] designed nanocomposites of CNTs/polyaniline and hybridized them with MXenes (Ti_3_C_2_T_x_) to obtain a composite electrode with superior gravimetric capacity and cyclic stability (>93% retention after 10,000 cycles). Studies on the mechanisms revealed that the surface capacitance storage was the major reason for its great rate potential, providing great opportunities for designing high-performance electrodes [117].

Li et al. [118] introduced N-doped CNT/MXene (Ti_3_C_2_T_x_)/polyacrylonitrile nanocomposite films as electrodes of supercapacitors, which were fabricated through vacuum filtration and electrospinning processes. These film electrodes with a high area-specific capacitance of 669.27 mF cm^−2^ and a high mass-specific capacitance of 446.18 F g^−1^ at 5 mV s^−1^ displayed suitable cycling stability with the retention of 90.9% after 4000 cycles, showing small capacity loss and good flexibility [118]. Despite the fascinating advantages of MXenes, such as significant volumetric capacitance, electrical conductivity, and hydrophilic properties, MXene-based electrodes may suffer from poor rate potential due to sheet restacking, particularly when the loading level is excessive and solid-state gels are employed as electrolytes [119]. Thus, MXenes were hybridized with CNTs to obtain fibers with helical structures, offering open spaces for rapid ion diffusion and obtaining fast electron transport. Accordingly, the designed fibers were applied in designing solid-state supercapacitors with gel electrolyte coating to show a volumetric capacitance of 22.7 F cm^−3^ at 0.1 A cm^−3^ with capacitance retention of 84% at a current density of 1.0 A cm^−3^ (19.1 F cm^−3^); these supercapacitors with mechanical robustness exhibited an enhanced volumetric energy density of 2.55 mWh cm^−3^ at a power density of 45.9 mW cm^−3^ [119]. Yang et al. [120] constructed MXene (Ti_3_C_2_)-CNT films and deposited them onto graphite paper through an electrophoretic deposition technique for supercapacitor electrodes. These electrodes with improved specific capacitance exhibited cycling stability (~10,000 cycles), thus displaying enhanced electrochemical performance [120].

**Figure 9:**
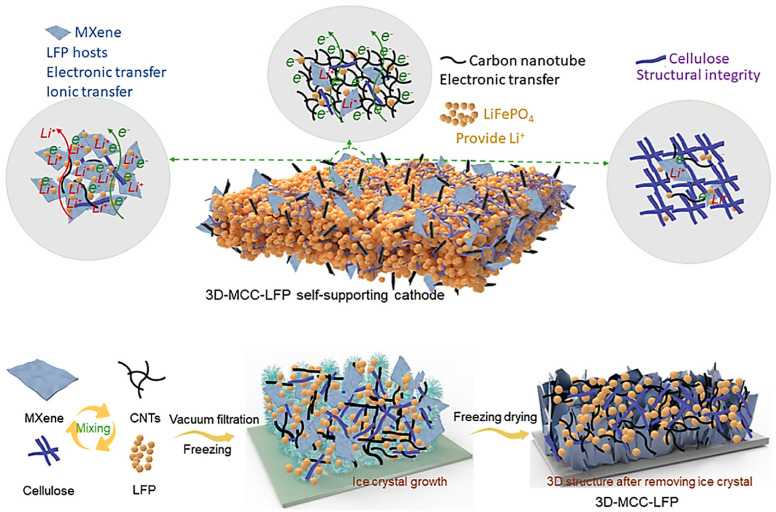
The preparative process of 3D MXene (Ti_3_C_2_T_x_)-CNT-cellulose-LiFePO_4_ (3D-MCC-LFP) cathodes. Adapted from Ref [37] with permission. Copyright 2022 Elsevier.

For improving the energy density of batteries, researchers have focused on the construction of cathodes with electroactive materials. However, irreversible Li^+^ consumption in full-cell configurations and poor structural integrity, along with low electronic/ionic transport are crucial challenges in this way. In one study, 3D MXene (Ti_3_C_2_T_x_)-CNT-cellulose-LiFePO_4_ cathodes were designed with improved LiFePO_4_ loading (120 mg cm^−2^) and enhanced electronic/ionic transport, providing better electrochemical performance, a significant capacity of 0.86 mAh cm^−2^ at 5 C, and a high retaining capacity of 1.45 mAh cm^−2^ after 500 cycles at 1 C (Figure 9) [37]. These cathodes exhibited an ultrahigh areal capacity of 19.2 mAh cm^−2^, and the introduced 3D-MXene-CNTs-cellulose-LiFePO_4_/SnO_2_ full-cell had a high areal capacity of 6.3 mAh cm^−2^ at 1.6 mA cm^−2^, offering MXene/CNT hybrid composites as promising materials in designing Li-ion batteries with high performance [37]. In addition, hydroxyl-functionalized Mo_2_C-based MXene nanosheets were fabricated through the simple removal of the Sn layer of Mo_2_SnC [121]. The prepared MXene with its hydroxyl-functionalized surface could suppress the shuttle effect of lithium polysulfides via robust interactions between Mo atoms on the surface of MXene and lithium polysulfides. For enlarging the specific surface area of final composites, CNTs were utilized into the Mo_2_C phase, improving the electronic conductivity and alleviating the volume change during charging/discharging processes. The robust surface-bound S in the hierarchical Mo_2_C-CNT host could lead to excellent electrochemical activity in Li-S batteries, providing a large reversible capacity (≈925 mAh g^−1^), even after 250 cycles at a current density of 0.1 C [121].

## 3. Conclusions and Perspectives

A wide variety of hybrid composites has been designed using MXenes and their derivatives because of their reducibility and hydrophilicity along with their large surface area, ease of functionalization, and unique mechanical/electrical properties. MXenes with their layered structures and chemical compositions have been hybridized with CNTs to obtain composites for catalysis, Li-ion batteries/supercapacitors, electrochemical capacitors, sensing, water treatment/pollutants removal, and EMI shielding applications. However, their electrocatalytic and biomedical potentials have been relatively less explored by researchers. MXene-CNT composites with significant electrical conductivity and strong mechanical features can be considered as attractive candidates for EMI shielding in electronic devices. The need for additional explorations on wearable sensors, high-rate electrochemical energy storage, soft robotics, EMI shielding films, and catalytic reactions is felt more than ever. Future explorations ought to focus on developing wearable and stretchable strain sensors with ultrahigh sensitivity and an adjustable sensing range using MXene-CNT composites. MXenes with good conductivity and electrochemical properties as well as abundant surface terminations and a large surface area are attractive candidates for designing novel composites with applicability in electronics, energy storage, catalysts/electrocatalysts, and pharmaceutics/biomedicine.

The density, specific surface area, and pore volume as well as the mechanical, electrical/electronic (e.g., electrical conductivity), and thermal (e.g., thermal conductivity, photothermal, and electrothermal) properties of MXene-CNT hybrids need more specific evaluations. The development of novel approaches of functionalization and hybridization along with comprehensive investigations for the industrial/commercial production of MXene-CNT composites should be considered. Transferring from the laboratory phase to industrial production as well as production adhering to green chemistry tenets and environmentally benign principles are very important in this field. Although promising results have been reported, their reproducibility along with the ability to be industrialized is equally crucial. Moreover, challenges regarding the oxidation of MXenes during production and their stability still need comprehensive evaluation. Furthermore, both experimental and theoretical studies can help to overcome these challenges, thus improving the properties of these composites.

## Figures and Tables

**Table 1:** Some selected examples of MXene/CNT composites with versatile applications.

Applications	MXene/CNT Composites	Advantages and Properties	Refs.
Flexible energy storage; electroanalytical chemistry; brain electrodes; electrocatalysis	MXene (Ti_3_C_2_T_x_) nanoflakes/multi-walled CNT on electrospun polycaprolactone fiber networks	Areal capacitance (30–50 mF cm^−2^). Highly enhanced rate function (14–16% capacitance retention at a scan rate of 100 V s^−1^). Suitable flexibility and tolerance against repeated mechanical deformation	[45]
Composite electrodes for high-rate electrochemical energy storage	MXene (Ti_3_C_2_)/CNTs composite	Significant capacitance (up to 130 F g^−1^) in organic electrolytes. Significant capacitance retention over a wide scan rate range of 10 mV s^−1^ to 10 V s^−1^. Supercapacitors at low temperatures	[46]
Li-ion capacitors	Nb_2_CT_x_ (MXene)-CNT electrodes	High volumetric energy density (50–70 Wh L^−1^). The lithiated graphite/Nb_2_CT_x_-CNT exhibited the highest gravimetric activity	[47]
Sodium-based energy storage devices	Porous MXene (Ti_3_C_2_)/CNT composite films	Excellent volumetric capacity of 421 mA h cm^−3^ at 20 mA g^−1^. Good rate performance. High cycling stability	[48]
Li-based batteries and hybrid capacitors	MXene (Ti_3_C_2_T_x_)-CNT composite	High energy and power density	[49]
Li-S batteries with a high sulfur loading	MXene (Ti_3_C_2_T_x_)/CNT sandwiches	Significant capacity of 712 mAh g^−1^; a sulfur loading of 7 mg cm^−2^. Superb cycling stability; 0.025% capacity decay per cycle over 800 cycles at 0.5 C	[2]
Li-S batteries	3D conductive CNT/MXene framework modified separator	The separator provided initial capacity of 1415 mA h g^−1^ at 0.1 C, with the capacity retention of 614 mA h g^−1^ even after 600 cycles at 1 C	[50]
Li-S batteries	CNT/MXene (Ti_2_C) nanocomposites	High density electrochemical energy storage systems	[51]
Dendrite-free sodium-metal electrodes	Fibrous hydroxylated MXene (Ti_3_C_2_)/CNT composite	Significant average Coulombic efficiency of 99.2%. No dendrite after 1000 cycles. Long lifespan over 4000 h at 1.0 mA cm^−2^ with a capacity of 1.0 mAh cm^−2^	[52]
High performance alkali ion batteries	Sandwich-like N-doped CNT@MXene (Nb_2_C) composite	Excellent electrochemical performance	[53]
High-performance Li-ion capacitors	MXene (Ti_3_C_2_T_x_)/CNT composite films	Remarkable energy density of 67 Wh kg^−1^ with good capacity retention of 81.3% even after 5000 cycles	[54]
High-rate sodium- and potassium-ion storage	MXene (Ti_3_C_2_T_x_)-CNT composite	High electrochemical features for sodium- and potassium-ion storage. The electrode with superb rate capability	[55]
Flexible microelectronic devices; supercapacitors	MXene (Ti_3_C_2_T_X_)-CNT composite	Good areal capacitance of 61.38 mF cm^−2^ at a current density of 0.5 mA cm^−2^	[56]
Hybrid supercapacitors	MXene (Ti_3_C_2_T_x_)/CNTs	Hydrogen ion aqueous-based hybrid supercapacitors. Significant energy density of 62 Wh kg^−1^. Excellent cycling stability	[57]
Supercapacitors	MXene/CNT@MnO_2_ composite film electrode	Significant specific capacity of 221 F g^−1^. High flexibility and good cycling stability	[58]
Supercapacitors	Porous MXene/CNT films	Superb cycling stability with a capacitance retention of 99.0% (20,000 cycles) at 100 A g^−1^	[59]
Supercapacitors	MnO_2_@MXene (Ti_3_C_2_T_x_)/CNT fiber electrodes	Outstanding cycling stability of 86.3% after 10,000 cycles and excellent capacitance of 371.1 F cm^−3^	[60]
Supercapacitors	MXene (Ti_3_C_2_T_x_)/multi-walled CNT electrodes	Areal capacitance of 1.93 F cm^−2^ was obtained, which was higher than pure Ti_3_C_2_T_x_ and its composites	[34]
Supercapacitors	Fe_3_O_4_-MXene (Ti_3_C_2_T_x_)-CNT electrodes	Good capacitive performance	[61]
Mechanically resilient and electrically conductive elastomer nanocomposites	MXene (Ti_3_C_2_T_x_)-CNT composite	Enhanced electrical conductivity. Improved mechanical properties	[62]
Electrochemical hydrogen evolution from seawater	Polyoxometalate-derived hexagonal molybdenum nitrides (MXenes) supported by boron, N co-doped CNTs	Remarkable electrochemical stability in environments with different pH values. Small over-potential of 78 mV at 10 mA cm^−2^ and Tafel slope of 46 mV per decade	[63]
Oxygen evolution and reduction reactions	Fe/Co-CNT@MXene composite	Excellent electro-activities. Significant specific capacity of 759 mA h g ^−1^ at a current density of 10 mA cm^−2.^ High durability cycling	[64]
Electrochemical performance; organic electrolytes	MXene (Ti_3_C_2_)/CNT composite	At 2 mV s^−1^, the capacitance values of 85 F g^−1^ and 245 F cm^−3^ could be achieved. Excellent rate capability and suitable cyclability. Enhanced capacitance	[65]
Sensing	Polydimethylsiloxane/MXene/CNT foam strain sensor	Enough conductive reliability and stability with great compressibility (~75%) and outstanding durability (>1000 cycles)	[66]
Multifunctional sensors	Cobalt (Co)@nitrogen (N)-CNT/MXene composite	High stability and tensile range. Flexible supercapacitors (great cycling stability of ~85,000 cycles with coulombic efficiency of ~99.7%)	[67]
Electrochemical sensor for the determination of capsaicinoid content	MXene/poly(diallyldimethylammonium chloride)-CNTs/*β*- cyclodextrin composite	The wide linear range was 0.1–50 μmol L^−1^, the low limit of detection (LOD) was ~0.06 μmol L^−1^, the recovery rate was ~84.00–125.60%	[68]
Electrochemical sensor for detection of ochratoxin A	MXene (Ti_3_C_2_)-multi-walled CNT composite	The concentration range was 0.09–10 μmol L^−1^ with LOD of 0.028 μmol L^−1^	[69]
Microwave absorption	MXene (Ti_3_C_2_T_x_)-CoNi@N-doped CNT composite	High surface areas (55.6–103.7 m^2^ g^−1^), moderate magnetism (19.8–24.6 emu g^−1^). Improved thermal oxidation stability (≥307 °C)	[70]
EMI shielding	CNT/MXene (Ti_3_C_2_)/ cellulose composite	The improved electrical conductivity was 2506.6 S m^−1^. EMI shielding effectiveness was 38.4 dB	[71]
Ultra-broadband electromagnetic wave absorption	MXene (Ti_3_C_2_T_x_)/ magnetic CNT composite	Enhanced electromagnetic wave absorption The minimum reflection loss of −51.98 dB (at thicknesses of 1.9 mm) and the maximum effective absorption bandwidth of 7.76 GHz (at thicknesses of 2.1 mm) could be achieved.	[72]
Improved electromagnetic wave absorption features	MXene (Ti_3_C_2_T_x_)-CNT composite	A minimal reflection loss of −52.56 dB (99.9994% electromagnetic wave absorption) in the X-band. High performance.	[73]
Broadband microwave absorption	MXene (Ti_3_C_2_T_x_)/CNT hollow microspheres	Remarkable microwave absorption properties. The maximum reflection loss was −40.1 dB The effective bandwidth was 5.8 GHz	[74]
Electromagnetic wave absorption	MXene (Ti_3_C_2_T_x_)-CNT nanocomposite	The minimum reflection coefficient reached −52.9 dB, ~99.999% absorption	[75]
EMI shielding films	Cellulose nanofibrils/multi-walled CNT microspheres intercalating MXene (Ti_3_C_2_T_x_)	Excellent mechanical robustness and durability	[38]

## Data Availability

Not applicable.
